# Electrospun Nanofibers of Natural and Synthetic Polymers as Artificial Extracellular Matrix for Tissue Engineering

**DOI:** 10.3390/nano11010021

**Published:** 2020-12-24

**Authors:** Mina Keshvardoostchokami, Sara Seidelin Majidi, Peipei Huo, Rajan Ramachandran, Menglin Chen, Bo Liu

**Affiliations:** 1 Laboratory of Functional Molecules and Materials, School of Physics and Optoelectronic Engineering, Shandong University of Technology, Xincun West Road 266, Zibo 255000, China; minak@sdut.edu.cn (M.K.); peipeihuo@sdut.edu.cn (P.H.); rajan@sdut.edu.cn (R.R.); 2 Interdisciplinary Nanoscience Center (iNANO), Aarhus University, DK-8000 Aarhus C, Denmark; sarasmajidi@inano.au.dk (S.S.M.); menglin@eng.au.dk (M.C.); 3 Sino-Danish College (SDC), University of Chinese Academy of Sciences, Beijing 100049, China; 4 Department of Engineering, Aarhus University, DK-8000 Aarhus C, Denmark

**Keywords:** electrospinning, scaffold, nanofibers, extracellular matrix, bioengineering

## Abstract

Many types of polymer nanofibers have been introduced as artificial extracellular matrices. Their controllable properties, such as wettability, surface charge, transparency, elasticity, porosity and surface to volume proportion, have attracted much attention. Moreover, functionalizing polymers with other bioactive components could enable the engineering of microenvironments to host cells for regenerative medical applications. In the current brief review, we focus on the most recently cited electrospun nanofibrous polymeric scaffolds and divide them into five main categories: natural polymer-natural polymer composite, natural polymer-synthetic polymer composite, synthetic polymer-synthetic polymer composite, crosslinked polymers and reinforced polymers with inorganic materials. Then, we focus on their physiochemical, biological and mechanical features and discussed the capability and efficiency of the nanofibrous scaffolds to function as the extracellular matrix to support cellular function.

## 1. Introduction

**Figure 1:**
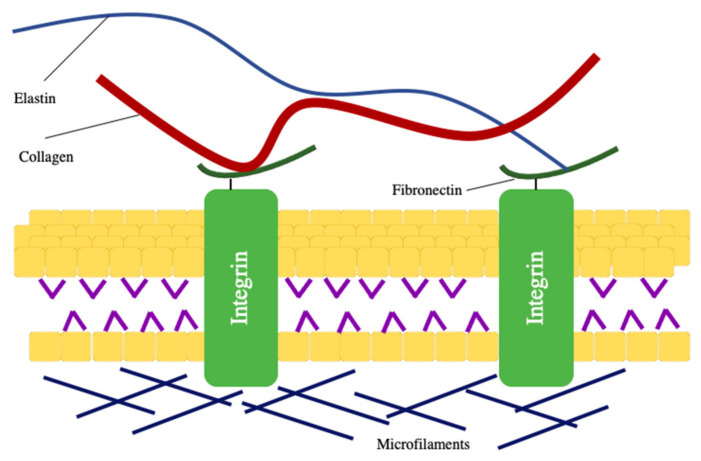
The structure and components of the extracellular matrix.

Tissue engineering concerns various research areas, and one of the challenges is to develop scaffolds to mimic the extracellular matrix (ECM). When attempting to build a suitable artificial scaffold, different criteria such as compounds, mechanical properties and structure must be considered Figure 1. Only when these criteria are optimized, the cells can function properly, such as proliferation, differentiation and migration [1,2,3]. Ideally, the components of engineered scaffolds should be in proximity to the native ECM, and they are often made of natural, biodegradable and biocompatible materials [4,5,6]. In terms of mechanical strength, scaffolds can withstand compressive and tensile stress by having fibril networks [7,8]. Such porous nanofibrous scaffolds with a higher surface to volume ratio are also similar to crosslinked porous collagen fibers (50–500 nm) found in the native ECM [9,10,11]. Hence, substantial effort has been devoted to producing nanoscale fibers to imitate the architectural structure of the native ECM [12,13,14].

**Figure 2:**
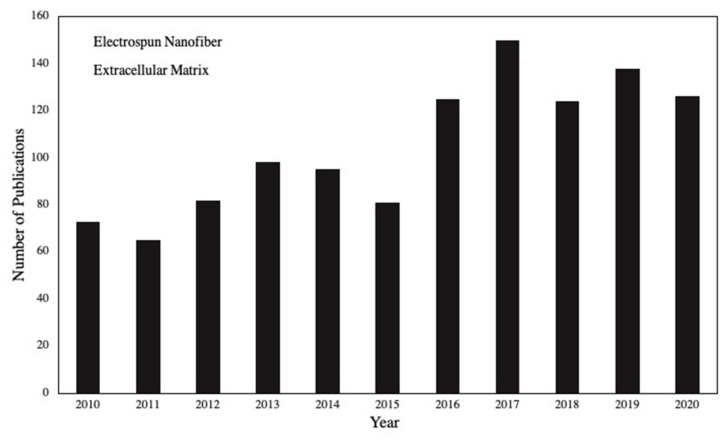
The number of published papers on electrospun nanofibers as an extracellular matrix.

Different polymers, whether natural or synthetic, have been electrospun to form the fibrous scaffold analogous to mimic ECM to support cell activities [15,16,17]. As shown in Figure 2 (keywords= electrospun nanofiber AND Extracellular matrix), scientists have combined different polymers, biomacromolecules or even inserted mineral materials to promote process capabilities, mechanical and biological mimicry of the artificial ECMs [18,19,20,21].

Most review papers regarding electrospun nanofibers focus on the application of the fibers in a particular tissue engineering field such as cancer, skin, bone, etc. In the present review, the impact of applying synthetic or natural polymers as artificial ECMs in the pure and modified forms in conjugation with other polymers, inorganic materials and crosslinking reagents was assessed. Initially, a short explanation of nanofibers and the electrospinning method, as the most considerable method for the synthesis of nanofibers, is defined. Then, fabrication and properties of five categories of electrospun composed polymers, including natural polymer-natural polymer, natural polymer-synthetic polymer, synthetic polymer-synthetic polymer, crosslinked polymers and polymer–inorganic materials-especially those applied as scaffolds, are described.

## 2. Application and Manufacture of Nanofibers

**Figure 3:**
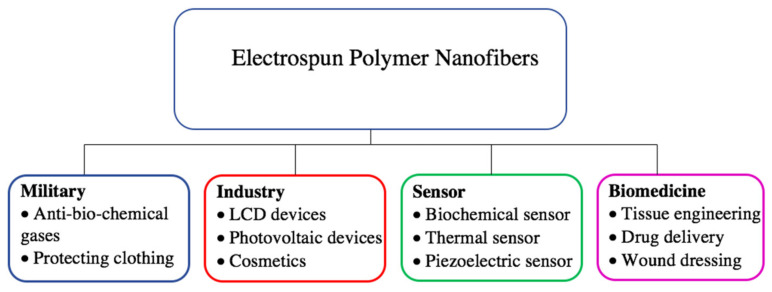
Applications of electrospun polymer nanofibers.

Nanofibers, due to their outstanding properties such as porosity, ease of synthesis, controlling of their composition, mechanical, and surface features, are distinct from other types of nanostructures [22,23,24]. Their applications, as shown in Figure 3, have been assessed in various fields, such as biomedicine, sensor, military and industry [25,26,27,28,29]. In the biomedical field, nanofibers have gained recognition in tissue engineering [30,31,32]. As most of the human organs and tissues are constructed of fibrous frameworks, nanofibers are ideal for mimicking and fabrication of artificial matrices that provide appropriate environments for cell functions [33,34,35]. Many biological assays have shown that nanofibers are extensively applicable as ECM scaffolds, as they obtain a vast surface area for cell adherence compared to bulk fibers [36,37,38]. Moreover, the architecture of nanofibers resembling the native ECM provides effective passage for oxygen and nutrition transportation [39].

Generally speaking, drawing, self-assembly, freeze-drying, phase separation, template synthesis and electrospinning techniques are the common strategies for creating nanofibers consisting of natural or synthetic polymers [40,41]. Each method has advantages and disadvantages; for example, manufacturing fibers with diameters less than 100 nm is highly difficult by applying the drawing method [42]. The self-assembly method, on the other hand, is time-consuming and often costly [43]. Although freeze-drying in terms of simplicity and expense was extensively used in the last two decades, the biggest challenge remains the lack of access to uniform porous fibers [44]. When using phase separation, the structure and pore size of the fibers are controllable, but similar to the template synthesis method, fabrication of long fibers is limited to few polymers [45,46]. While electrospinning is known as the most eminent technique, it is still challenging to provide nanofibers at an industrial scale [47].

## 3. The Electrospinning Principle and Processing

In recent years, the electrospinning technique has shown the merit of manufacturing-oriented electrospun nanofibers that are architecturally analogous to the nanofibrous framework of the ECM in the biological environment [48,49,50]. This method is versatile, facile, rapid, efficient and is dependent upon the ejection of a polymer solution driven by a high voltage potential between a positively charged needle and a grounded collector [51,52,53]. By evaporation of the solvent, the generated fibers from nanometer to micrometer ranges are collectible. Thereby, a high voltage supply, a collector plate and a syringe as spinneret are the three main units in an electrospinning setup [54,55].

Different polymers, whether natural, artificial or a combination of them or other materials, were examined as components of electrospun nanofibers [56,57,58,59]. Three key parameters control the morphology and diameter of fabricated fibers [60,61]. The first element is the viscosity that is directly affected by the mass of polymeric solutions and molecular weight of polymers. On one hand, too low viscous solutions produce beaded fibers, while on the other hand, too high viscous solutions lead to the inability to spin [62]. Hence, preparing solutions with proper concentrations is a key parameter. The other parameters are surface tension and polymer molecular weight. Solutions with high surface tension and low conductivity lead to bead formation [63,64], while higher molecular weight polymers tend to form micro ribbon fibers [65,66]. To improve the performance of polymers with low molecular weight, integration of higher molecular weight polymers can be efficient [67].

Moreover, as ambient factors, the function of temperature and humidity influence the fiber morphology as well [68,69]. Thus, in order to obtain a stable condition during the spinning process, an environmental chamber is ideal [70]. Based on the above explanation, for creating suitable fibers, solution properties, electrospinning parameters and environmental conditions are highly determinative [71,72]. Thus, nanofibrous frameworks that are architecturally analogous to the native ECM can be prepared by optimizing various conditions.

## 4. Nanofibers as Scaffolds

Nanofibrous forms of various polymers have been introduced as artificial ECMs [10,11,12,13,14,15,16,17,18,19,20,21,22,23,24,25,26,27,28,29,30,31,32,33,34,35,36,37,38,39,40,41,42,43,44,45,46,47,48,49,50,51,52,53,54,55,56,57,58,59,60,61,62,63,64,65,66,67,68,69,70,71,72,73]. This substantial attention to polymeric materials is due to the surface functionalization possibility, tunable diameter and ease of combination with different materials [74]. Chitosan [75,76,77], collagen [17,18,19,20,21,22,23,24,25,26,27,28,29,30,31,32,33,34,35,36,37,38,39,40,41,42,43,44,45,46,47,48,49,50,51,52,53,54,55,56,57,58,59,60,61,62,63,64,65,66,67,68,69,70,71,72,73,74,75,76,77,78,79], gelatin [80,81] and silk [82,83,84] are the most cited natural polymers used as electrospun nanofibrous scaffolds, while polycaprolactone (PCL) [85,86,87], poly-lactide acid (PLA) [88] and poly(lactic-co-glycolic acid) (PLGA) [89] are well-known synthetic polymers. In order to compensate for the deficiency of the polymers in bioactive cues and to create better scaffolds for cells, researchers have combined them with biomacromolecules, inorganic materials or crosslinked them with different materials. Some of these scaffolds are summarized in Table 1 and described below.

### 4.1. Natural Polymer-Natural Polymer Composite Nanofibers

**Figure 4:**
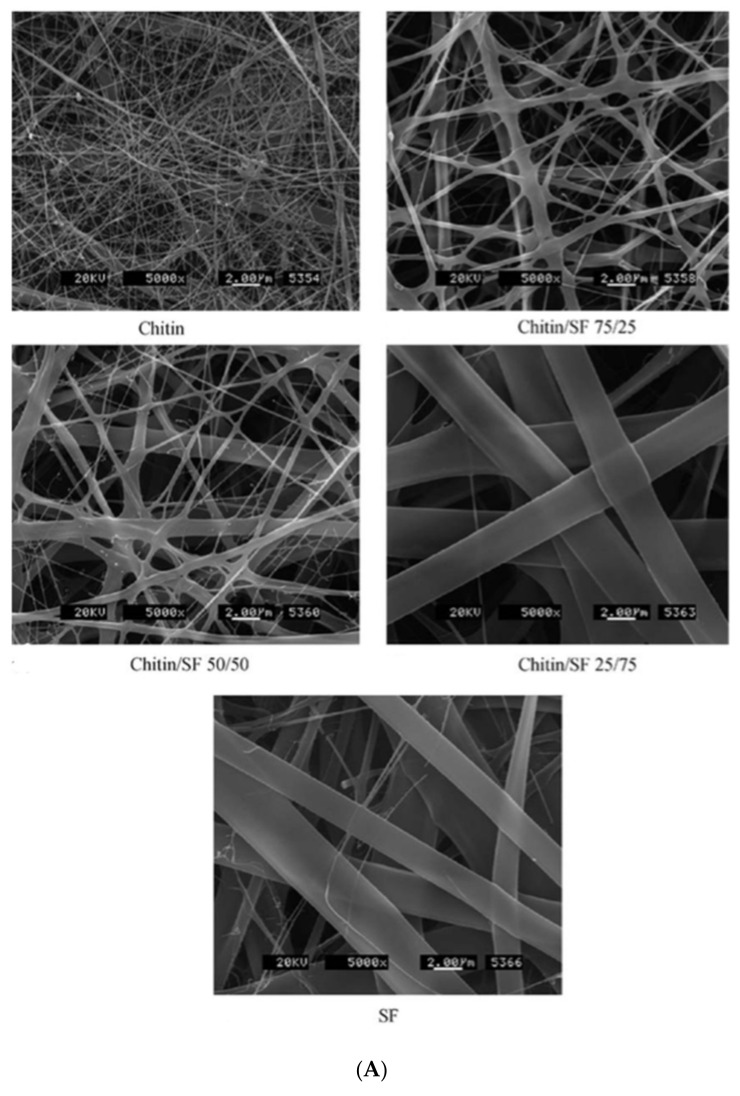

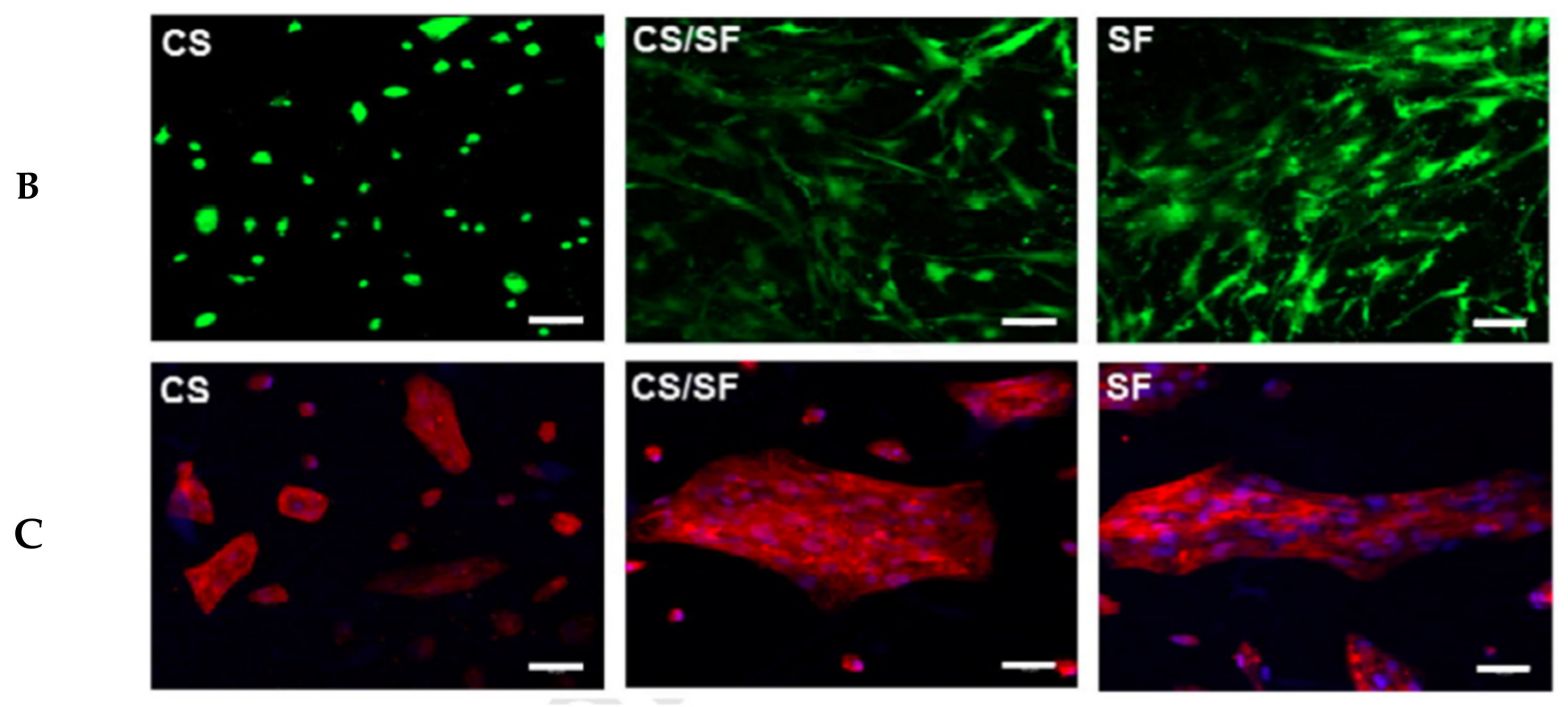
(**A**) SEM pictures of SF/chitin nanofibers at magnification 5000×. Reproduced from reference [82] with a permit from Elsevier. (**B**) Live-dead [88] and (**C**) phalloidin-DAPI staining [91] of human bone marrow stem cells seeded on CS fibers, SF and CS/SF nanofibers after 72 h. Scale bar is 100 μm in (**B**) and 50 μm in (**C**). Reproduced with permission from Elsevier.

Natural polymer nanofibers, due to eminent features like biodegradability and biocompatibility, are noteworthy materials in biological environments [110]. Ideally, they can create scaffolds similar to natural ECMs in design and structure that allow cells to perform vital tasks including signal transmission, proliferation, differentiation etc. [111,112]. Chitin and its over 50% deacetylated derivative, chitosan, are applicable natural polysaccharides that are widely utilized as scaffolds. Beyond special biological properties, their high hydrophilicity features cause deformation [113,114]. In order to have a more robust composite, combinations with other materials are indispensable. For example, novel ECM scaffolds were produced from chitin/silk fibroin (chitin/SF) nanofibers [82]. Viscosity evaluation of the nanofibrous composite proved that chitin and silk fibroin are completely miscible. In addition, morphology and structure analysis demonstrated that by increasing the chitin quantity, the diameter decreased from 920 nm to 340 nm Figure 4A. Generally, nanofibers with lower diameters will be created from polymers with greater polarity during electrospinning. Furthermore, scaffold cytocompatibility behavior on normal human epidermal fibroblast (NHEF) and keratinocyte (NHEK) cells was evaluated. For this reason, the cells were dispersed on the scaffold. The obtained data varied among different combinations of chitin and silk fibroin. The matrix comprised of 75% chitin and 25% silk fibroin, due to its three-dimensional structure and desirable NHEF and NHEK adherence and spreading, was found to be the best candidate as ECM. In another study, Lai et al. [91] synthesized electrospun mats of chitosan (CS), silk fibroin and 1:1 mass ratio of a chitosan/silk fibroin composite and determined their physicochemical characteristics by scanning electron microscope (SEM), Fourier-transform infrared spectroscopy (FTIR), and X-ray diffraction (XRD). The performance of the mats for culturing of human bone marrow stem cells was compared. The obtained outcomes of alkaline phosphate activity, Alizarin Red staining, and expression of osteogenic marker gene analysis indicated that mixing the mentioned polymers not only preserved the osteogenic characteristic of chitosan but also increased proliferation and differentiation of the cells Figure 4B,C. Therefore, chitosan/silk fibroin nanofibers are suitable potential scaffolds in bone regeneration. With the purpose of introducing proper scaffolds for retinal cells, Noorani et al. [92] reported nanofibrous scaffolds comprised of a chitosan/gelatin blend with mean fiber diameters of 180 nm. They claimed that the addition of gelatin to chitosan promoted its hydrophilicity and degradation along with decreasing mechanical properties. Samples with higher gelatin percentage showed lower tensile strength and Young’s modulus. The maximum tensile strength was reported 6.93 ± 0.63 MPa for gelatin/chitosan (50/50) scaffold, while the scaffold with 30% chitosan had a maximum strength of 3.51 ± 0.45 (*p* < 0.05). Young’s modulus of the prepared gelatin/chitosan scaffolds with 70/30 and 50/50 ratios were obtained as 1.05 and 2.24 MPa, respectively. In continuation, chitosan/gelatin nanofibers were applied as scaffolds for retinal pigmented epithelial (RPE) cells, where a 3-(4, 5-dimethylthiazol-2-yl)-2,5-diphenyltetrazolium bromide (MTT) test was applied to evaluate cell viability. The captured SEM images showed the RPE cells were properly attached to the substrate. Thus, inserting gelatin into chitosan fibers developed excellent scaffolds in regards to mechanical and biological characteristics.

In order to achieve effective scaffolds for blood vessel tissue engineering, Guibo et al. [90] combined silk fibroin with gelatin. The addition of gelatin to silk fibroin in different concentrations exhibited better spinnability and viscosity for the silk fibroin nanofibers. The resultant electrospun nanofibers showed better mechanical properties than pure silk fibroin. The promotion in mechanical properties is due to more intermolecular hydrogen bonding in the composite and an increase in ß-sheet structure compared to pure silk fibroin. Breaking hardness, strain at break, average fiber diameter, percent porosity and the average diameter of homogeneous nanofibers were reported as 1.6 MPa, 7.6%, 89.2 nm, 87% and 89.2 nm, respectively. After characterization, the authors investigated the viability of human umbilic vein endothelium cells (HUVECs) and mouse fibroblasts when using the prepared fibers. By considering the cell culture responses that demonstrated proper adherence and increase of HUVECs and mouse fibroblasts on the scaffold, they concluded that the nanofibers have a tremendous potential to be used as a natural ECM for blood vessel engineering.

Among different natural polymers, remarkable efforts have been put into collagen-containing nanofibrous materials, as the native ECMs are principally made of collagen. However, the application of collagen, owing to high price, low melting point, and fusion of nanofibers in aquatic media, is restricted. Wang et al. [115] endeavored to utilize electrospun collagen peptide/chitooligosaccharides composite membranes as an ECM for human skin fibroblasts. Their research consisted of three major parts. First, fish scale collagen peptide (FSCP) with low molecular weight was blended with chitooligosaccharides (COS) with a 2:1 mass ratio. In this step, polyvinyl alcohol (PVA) was applied to improve fiber-forming, and the nanofibers were prepared using the electrospinning technique. Second, a microstructure analysis was fulfilled, where SEM images showed nanofibers with diameters between 50 nm and 100 nm. Finally, they investigated the antibacterial activity of the electrospun membranes against Gram-positive *Staphylococcus aureus* and Gram-negative *Escherichia coli* and found the antibacterial activity against *S. aureus* was higher than against *E. coli*. Furthermore, MTT analysis was applied to evaluate the potential of the membranes for culturing of fibroblasts. The obtained results indicated that FSCP/COS nanofibers are good support for fibroblast proliferation. In another study, Noh et al. [116] introduced bacterial cellulose-collagen composite scaffolds in different ratios (1:1, 3:1, 5:1) to assess their impact on human mesenchymal stem cells (hMSCs). In comparison to pure collagen scaffolds, the composite presented better physical stability and higher water uptake by increasing the bacterial cellulose content. Gene and protein analysis of three weeks of cultured umbilical cord blood-derived mesenchymal stem cells (UCB-MSCs) on the composites showed that among different ratios, the bacterial cellulose-collagen composite in the 5:1 ratio was the most impressible substrate. In vivo studies were performed on mice and demonstrated that there were many transplanted cells in the mats.

In this section, we compared nanofibrous scaffolds composed of chitin/silk fibroin, chitosan/silk fibroin, chitosan/gelatin, collagen peptide/chitooligosaccharides and cellulose/collagen. In all studies, the material composition leads to promotion in mechanical and biological features in comparison to scaffolds made of one polymer. In the case of silk fibroin, by raising the quantity, the diameter of the fibers increased. Therefore, it should be considered that nanofibers with lower diameters will be created from polymers with greater polarity.

Combining natural polymers is a simple strategy to provide suitable substrates for cell activities. However, their main disadvantages include quick biodegradation, poor processability and weak mechanical characteristics [117,118]. Combining natural polymers with synthetic polymers, such as PCL, polyurethane and polyaniline, is another strategy to produce artificial ECMs.

### 4.2. Natural Polymer-Synthetic Polymer Composite Nanofibers

Synthetic polymers are inexpensive and can be fabricated into various porous structures, which, in combination with natural polymers, provide optimal support for cell attachment and proliferation [119,120]. For example, Vatankhah et al. [93] electrospun a polymeric solution of hydrophilic polyurethane called Tecophilic (TP) and gelatin to overcome vascular regeneration challenges. They observed that the (TP(70)/gel(30)) composite scaffold prevents thrombogenicity due to the hydrophilic properties of TP, while gelatin improves adhesion capacities of vascular smooth muscle cells (SMCs). Furthermore, the nanofibrous scaffold was sufficiently durable to tolerate cyclic-loading like native blood vessels.

Tubular scaffolds made of poly (l-lactic acid) (PLLA) and various concentrations of gelatin were prepared in order to mimic a blood vessel supportive platform [121]. The aligned nanofibers were analyzed with FTIR and SEM. By increasing the gelatin concentration, the hydrophilicity of the scaffold, as well as SMCs attachment and proliferation, improved. It is believed that the morphology of aligned fibers assists cells to orient their long axis. Gu et al. [96] investigated the mechanical and biological features of aligned and conductive nanofibrous scaffolds by blending chitin with polyaniline as a conductive polymer (Chi/PANi) in order to directionally guide the human dermal fibroblast cells (HDFCs) during culture. Random and aligned fibers were prepared using electrospinning equipped with a drum collector. The alignment of nanofibers in tissue engineering is important for increasing the directional guidance of cells. Electrostatic and rotational interactions of the drum collector produced aligned Chi/PANi nanofibers. The width of the aligned nanofibers was reported to be 49% smaller compared to random nanofibers, while the electrical conductivity of aligned Chi/PANi nanofibrous scaffolds was ~91% higher compared to random nanofibers. After one week, the viability of the cells on the surface of the aligned nanofibers was ~2.1 times greater than on the random nanofibers. In conflict with Gu et al. [96], Guo et al. [95] believe that electrospinning with a rotating disk collector with high-speed decreases nanofibers alignment. Therefore, Guo et al. used two parallel plate collectors to synthesize aligned PCL/gelatin fibrous scaffolds to achieve an oriented morphology similar to the native ECM. While the mean diameter of both aligned and random fibers was around 330 nm to 370 nm, the aligned fibers resulted in better mechanical properties, cell growth, and cell proliferation.

PCL is sufficiently biocompatible and biodegradable to be applied in tissue engineering, although its hydrophobic nature restricts its application [122,123,124]. In order to solve this challenge, Anjum et al. [125] constructed nanofibrous scaffolds of PCL and gelatin. The scaffolds were comprised of PCL mixed with gelatin (PCL-bGE), PCL covered with gelatin (PCL-cGE), and PCL conjugated with arginylglycylaspartic acid (RGD). Conjugation of polymers with RGD leads to significant improvement in cell proliferation because RGD exists in ECM proteins. Seeded human skin-derived precursor cells (hSKPs) on the nanofibers showed a higher amount of DNA after 28 days on the PCL-RGD and PCL-gelatin composite scaffolds compared to the pure PCL scaffold. Their findings from in vivo tests indicated the production of collagen III was enhanced on all scaffolds except for the PCL-cGE scaffold. Therefore, PCL-bGE nanofibers have a greater potential to be applied as scaffolds for wound healing compared to PCL-cGE. Recently, Sharifi et al. [94] hypothesized that carboxymethylation of chitosan (CMC) and its combination with PCL could create scaffolds similar to the native bone ECM. In this context, they fabricated two different nanofibrous scaffolds by electrospinning: polycaprolactone/chitosan (PCL/CTS) and polycaprolactone/carboxymethyl chitosan (PCL/CMC). SEM images showed that the average fiber diameter of PCL/CMC fibers was smaller than that of PCL/CTS fibers. Moreover, undesirable fibers disappeared after carboxymethylation because the charge density and viscosity of the electrospinning solution were adjustable. Seeding of human osteoblast cells (MG63) on both types of fibers proved promotion in cell proliferation on PCL/CMC compared to PCL and PCL/CTS.

Combining drug delivery systems with tissue engineering is a strategy for directly transferring drugs to intended sites to increase their efficacy and reduce side effects. For instance, various compositions of polycaprolactone/collagen (PCL/Coll) electrospun nanofibers coated with transforming growth factor-beta (TGFβ) were prepared for tuning myofibroblast differentiation. This growth factor induces the transition from fibroblasts to myoblasts, which reduces the wound size during wound healing. Gene expression and immunofluorescence imaging exhibited TGFβ1 loaded PCL/Coll (40%/60%, *w/w*) had the optimum cell viability and myofibroblast differentiation capacity [126]. In another study, Molas and Chen fabricated injectable core-shell nanofibers for delivery of hMSCs as therapeutic cells. Here, the synthetic polymer ((poly(lactide-co-ε- caprolactone))) (PLCL) was chosen as a core, and a hydrogel (gelatin–methacrylate (Gel–MA)) was used as the shell. Two factors affected the mean fiber diameter: enhancement in the concentration of PLCL solution and raising the speed of electrospinning of the core solution. The core-shell system assisted the proliferation of hMSCs and, besides, favored injection of cellularized mats [127].

In this section, it was found that the composition of natural and synthetic polymers displayed better mechanical durability, wettability and cell attachment in comparison to scaffolds composed of one component. Beyond comparing the potential of the scaffolds composed of natural and synthetic polymers for cell growth, the effect of nanofiber alignment on cells was considered. Aligned fibers aid cells to orient their long axis, thereby making them more appropriate for cell growth and proliferation. Applying polymers such as hydrophilic polyurethane and polyaniline improved hydrophilicity and conductivity properties, respectively. An increase in the concentration of PCLC-caused fibers with a larger diameter. Although desired results were obtained by combining natural and synthetic polymers, a variety of artificial ECMs constructed of synthetic polymers have provided ideal environments for cells, as described in the following section.

### 4.3. Synthetic Polymer-Synthetic Polymer Composite Nanofibers

Synthetic polymers verified by the Food and Drug Administration (FDA) are suitable for tissue engineering. However, the hydrophobicity of synthetic polymers should be compensated by the addition of hydrophilic components such as poly-ornithine, polyethylene oxide (PEO) and PVA. Nylon 6 (N6), owing to the chemical structure resemblance to collagen and stability in the human body, has attracted much attention, especially in bone tissue engineering. For instance, nanofibers made from the deposition of PVA on N6 using a hydrothermal method were applied as scaffolds for pre-osteoblast MC3T3-E1 cells. The obtained results clarified the presence of PVA stimulated the crystalline conformation of N6 and increased hydrogen bonding interaction. PVA enhances the wettability of the N6 mat, leading to well-attached MC3T3-E1 cells [128]. In a new approach to improve bone tissue regeneration, Fu et al. [129] optimized the surface properties of PLLA nanofibers with ECM. At first, MC3T3-E1 cells were cultured and allowed to be grown on the electrospun nanofibers in order to have an ECM deposit on the nanofibrous scaffold. After two weeks, cellular components were removed by decellularization. Cell adhesion and osteogenic differentiation of cells were remarkably improved compared to PLLA nanofibers without ECM. In another study, Xu et al. [130] offered simultaneous delivery of fibroblast growth factor 2 (FGF-2) and connective tissue growth factor (CTGF) from silk fibroin/PLCL-PEO coaxial electrospun fibers. FGF-2 is a fundamental factor for the proliferation of stem cells, while CTGF enhances fibrogenesis. The combination of FGF-2 and CTGF reinforces the proliferation of MSCs. In terms of spatiotemporal release of FGF-2 firstly and gradually distribution of CTGF, they embedded CTGF inside silk fibroin as the core and dispersed FGF-2 on PLCL- PEO as the shell. It is worth mentioning that applying the hydrophobic PLCL as a barrier slows down the diffusion of CTGF, whereas adding the hydrophilic PEO accelerates the release of FGF-2. They analyzed the release of CTGF and FGF-2 by ELISA and found the burst release of FGF-2 and the sustained release of CTGF efficiently enhanced the differentiation of MSCs in vitro. Conductivity, biological activities, and surface properties are critical factors in nerve tissue engineering [131]. Tian et al. [97] proposed aligned nanofibers of poly (lactic acid) and poly (pyrrole) with electrical conductivity in order to introduce a convenient scaffold for nerve regeneration applications. For compensation of electrical deficiency, poly (pyrrole), as a conductive polymer, was blended with poly (lactic acid). Surface hydrophilicity was improved by coating the composite with poly ornithine. Evaluation of neuronal differentiation of PC12 cells demonstrated appropriate results, even in the absence of electrical stimulation. Aligned fibers displayed better cell proliferation, and the combination of poly (lactic acid) with poly (pyrrole) decreased the fiber diameters. However, it did not increase the biocompatibility of the scaffold.

**Figure 5:**
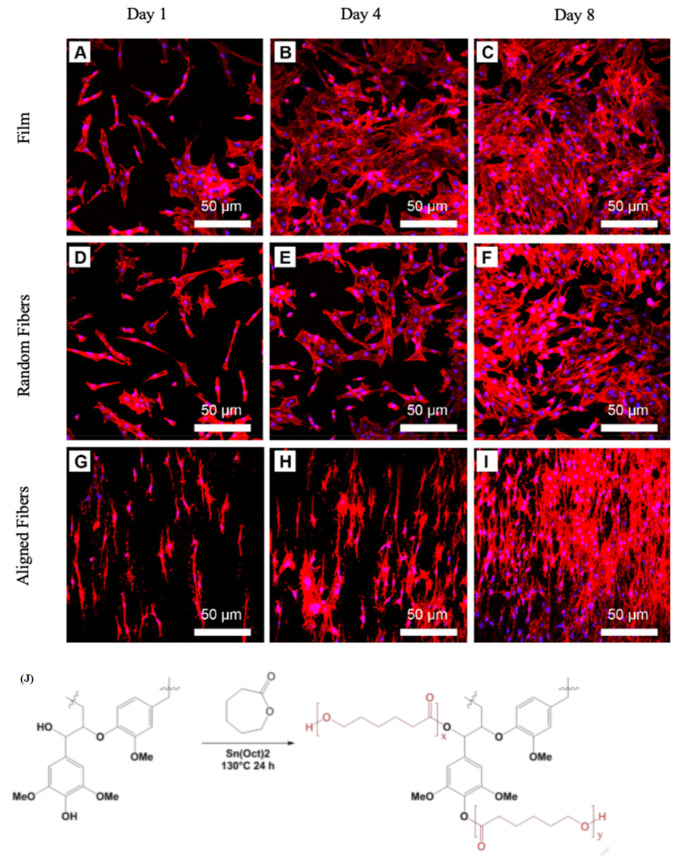
**(A–I**) Distribution and cell shape on poly(lactic-co-glycolic acid) (PLGA)/polycaprolactone (PCL) scaffolds by CLSM observation. Red color indicates actin. Blue color indicates nuclei. Duplicated from reference [98] with a permit from Elsevier. (**J**) synthesis of lignin-PCL copolymers by solvent-free polymerization. Duplicated from reference [99] with a permit from Elsevier.

High-stress tolerance in primary implantation is the crucial parameter in meniscus tissue engineering. For this purpose, Gao et al. [132] prepared electrospun random and aligned fibers from decellularized meniscus extracellular matrix (DMECM) (obtained by a novel centrifugation method) and PCL. On one hand, pure DMECM fibers were fragile, so it was essential to combine them with a polymer to promote their mechanical features. From another point of view, the hydrophilicity of PCL should be enhanced for tissue engineering applications. FTIR analysis confirmed that the two materials were properly combined. DMECM/PCL fibers indicated good mechanical properties and hydrophilicity for cell attachment. Furthermore, similar to the human meniscus, the tensile moduli of the aligned fibers were in the range of 132.27 MPa to 331.40 MPa. Zhong et al. [98] focused on creating scaffolds for long thin spindle-shaped smooth muscle cells. They built random, aligned and film membranes of PLGA/PCL composites. The width of random nanofibers was estimated to be around 643 nm, while for the aligned nanofibers, created using an electrospinning instrument with a rotation speed of 100 rpm, the obtained diameter was around 554 nm. The amount of human vascular smooth muscle cells (HVSMCs) increased over time on all three types of scaffolds. Evaluation of cell morphology on the film and random fibers after one day showed that cells were mainly spindle-shaped, but their morphology changed to stellate-shaped over time. In the case of the aligned scaffolds, the spindle-shaped cells did not alter with time Figure 5a–i. Another study by Wang et al. [99] focused on making antioxidant scaffolds to prevent neuron cells from oxidant stress-related injuries. The nervous system is vulnerable to reactive oxygen species and high amounts of unsaturated fatty acids. Lignin, as an antioxidant reagent, was combined with PCL via solvent-free ring-opening polymerization from ε-caprolactone Figure 5j. Subsequently, PCL/lignin-PCL nanofibers were prepared using electrospinning. Oxidative tests were carried out by exposing cultivated scaffolds with Schwann cells to hydrogen peroxide. They observed that lignin-containing scaffolds were properly protecting the cells from oxidative stresses related to the free radical scavenging property. Thus, the authors highly recommend nanofibrous PCL-lignin copolymer with convenient mechanical, biocompatible and antioxidant properties for nerve regeneration.

Among the applied synthetic polymers, nylon 6 (N6), owing to chemical structure resemblance to collagen, had attracted much attention. All the mentioned papers demonstrated that synthetic polymer nanofibers should be modified to be applicable in tissue engineering. The most significant issue is wettability, which can be improved by adding materials, such as poly-ornithine, polyethylene oxide, polyvinyl alcohol or decellularized ECM. Furthermore, all the results showed that among films, aligned and random nanofibers, aligned nanofibers highly support cells and help cells to orient their long axis, thereby increasing cell growth, proliferation and mechanical properties. Loading growth factors on the nanofibers considered an applicable method to improve cell proliferation. Lignin, as an antioxidant reagent, was employed to prepare PCL-lignin scaffold and properly protected the cells from oxidative stresses related to the free radical.

Generally, beyond the composition of polymers, crosslinking of hybrid polymers is a strong approach to engineer nanofibers and boost their stability and mechanical characteristics [133,134,135,136]. The next section is dedicated to crosslinked nanofibers used as scaffolds.

### 4.4. Nanofibers of Crosslinked Polymers

**Figure 6:**
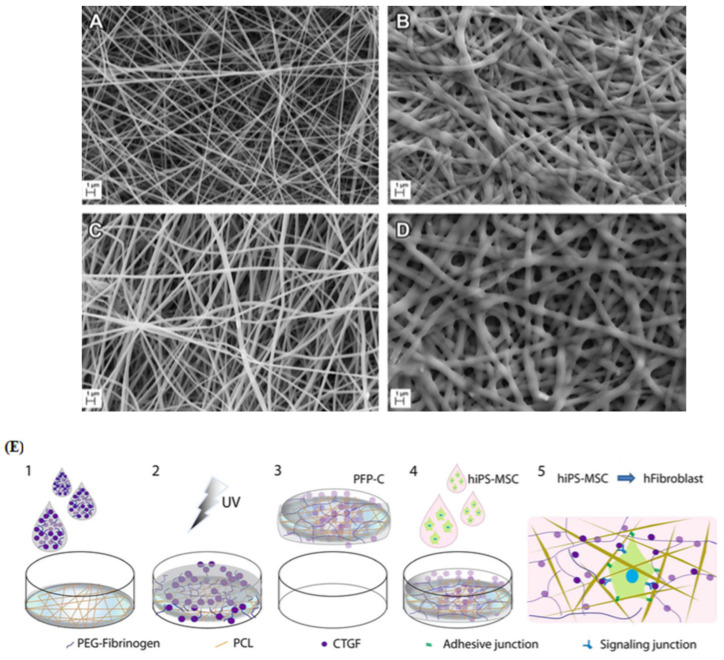
(**A**–**E**) SEM images of (**A**) pure gelatin, (**B**) crosslinked gelatin, (**C**) rat decellularized brain extracellular matrix (dBECM)-gelatin and (**D**) crosslinked dBECM-gelatin mats (scale bar: 1 μm). Reproduced from reference [142] with a permit from Elsevier. (**E**) Illustration of synthesis procedure for the PFP-C nanocomposite and its effect on hiPS-MSCs. (1) fibrinogen (PF) mixture containing connective tissue growth factor (CTGF) was subjoined to polycaprolactone (PCL) mesh; (2) UV irradiation for crosslinking PF; (3) PFP-C composites were created; (4) hiPS-MSCs were cultured on the scaffold; (5) fibrogenesis process synergistically promoted by the adhesive motif on PFP and the signaling induction of CTGF. Reproduced from reference [150] with permission from Nature.

Chemical crosslinking generates covalent connections between polymeric fibers [137]. Crosslinking processes for fibers are usually categorized into in situ electrospinning and post-spinning crosslinking [138,139]. Genipin (GP), glyoxal, glutaraldehyde (GA) and 1-ethyl-3-(3-dimethylaminopropyl) carbodiimide (EDC)/N-hydroxysulfosuccinimide (NHS) are widely used crosslinkers for gelatin in tissue engineering. Toxicity of crosslinker residues and alteration in the main morphology of gelatin nanofibers are general problems in chemical cross-linking [140,141]. Baiguera et al. [142] utilized GP as a crosslinker reagent for gelatin nanofibers. As explained in Section 4.3, Gao et al. [132] presented electrospun random and aligned fibers from decellularized meniscus extracellular matrix/PCL. Similarly, Baiguera et al. [142] modified the surface of electrospun crosslinked gelatin scaffolds with rat decellularized brain extracellular matrix (dBECM) for neural tissue engineering. First, dBECM powders were sonicated for 10 minutes in a solution containing acetic acid and deionized water. Then, gelatin powder was added, and the mixture was poured into a syringe for the preparation of nanofibers using the electrospinning method. The crosslinking procedure was carried out by soaking the electrospun mat in a GP solution. They deduced that the uniform, bead-free and fibrous structure did not change by applying a crosslinking procedure, but the mean diameter of the fibers was enhanced Figure 6a–d. Angarano et al. [143] presented nonwoven crosslinked gelatin nanofibers (CGN) and laminating CGN by 3D microextrusion of PCL fibers. Crosslinking of gelatin was performed by mixing a glyoxal solution, as a nontoxic and fluorine free crosslinking reagent, with the gelatin solution prior to electrospinning. They found the average diameter of nanofibers enhanced to 680 nm during electrospinning, which was due to the crosslinking process and an increase in viscosity of the gelatin solution. CGN nonwovens and non-crosslinked PCL nonwovens were combined by heating above the PCL melting temperature. In contrary to gelatin and CGN, the PCL/CGN system was mechanically and biologically convenient to be applied as ECM.

Nagarajan et al. [144] synthesized gelatin electrospun scaffolds crosslinked by GA and then loaded chlorohexidine as an antibiotic drug on the mats for wound dressing. They found that the drug-loaded mats were highly active against *E. coli* and *S. epidermidis* at pH 7–8. Biocompatibility analysis was performed by using keratinocytes and fibroblasts, which indicated high biocompatibility.

Agheb et al. [100] modified the surface of gelatin using tyrosine and 1, 2, 3-triazole rings according to cartilage-type tissue engineering. After the electrospinning process, interconnections between the fibers occurred by crosslinking the fibers with GA and EDC/NHS. In addition to antimicrobial and anti-viral properties, 1, 2, 3-triazole geometrically imitates natural peptides, while tyrosine can absorb antigens to polymers. Although GA is known as a cytotoxic reagent, its advanced manner as a crosslinking reagent in low concentrations, especially in tissue engineering, has been approved. Surface morphology analysis exhibited a remarkable decrease in the network pore size by the addition of tyrosine into the gelatin scaffold. Furthermore, chemical crosslinking of the modified gelatin by EDC/NHS or GA caused a reduction in porosity. In vitro chondrocyte culture displayed that the EDC/NHS crosslinked mat is more appropriate as a scaffold in cartilage engineering compared to pure gelatin, modified gelatin and GA crosslinked gelatin. In a study by Tonsomboon et al. [145], the authors reinforced electrospun gelatin nanofibers with alginate hydrogels to imitate the native corneal ECM. When creating a corneal ECM, a balance between mechanical and optical properties should be considered. As the corneal ECM is constructed mainly of water, alginate hydrogels, which are structurally similar to glycosaminoglycans (GAGs) in the corneal ECM, were applied to fortify the scaffold. Even though the mechanical features were improved by crosslinking the hydrogel in an EDC/NHS solution, a decrease in transparency limited their applications.

GAGs are one of the fundamental components in the ECMs, which affect cell attachment and proliferation [146,147]. Pezeshki et al. [148] combined chondroitin sulfate as GAG with gelatin nanofibers crosslinked with EDC. Furthermore, the authors used response surface methodology to optimize the electrospinning process. They realized the mean fiber diameter decreased by enhancing the voltage and the proportion of chondroitin sulfate. Additionally, HDFCs fully cellularized on the scaffold.

In a study by Gomes et al., GA was used as a crosslinking reagent for three categories: gelatin as a polymer, PCL as polyester and chitosan as a polysaccharide [101]. After physiochemical characterizations, in vitro tests were fulfilled by seeding human fetal fibroblasts (HFFF2) on the scaffolds. The results revealed cell proliferation and adhesion on all scaffolds. However, the cell viability on the crosslinked gelatin scaffold decreased after 48 h, which could be related to GA residuals. In in vivo tests, the crosslinked chitosan scaffold functioned better than the gelatin scaffold in the treatment of skin wounds. To enhance mechanical features and dominate shortcomings of pure polymers, Nagiah et al. [149] suggested crosslinking of coaxially electrospun nanofibers. In the coaxial electrospinning system, poly (3-hydroxybutyric acid) (PHB) and gelatin were selected as the core and shell, respectively. Crosslinking was accomplished by immersing the nanofibers in a GA solution. As previously observed, when using gelatin-based biomaterials, the scaffold successfully reinforced adherence and growth of HDFs and keratinocytes. For mimicking dermal ECM, Bhowmick et al. [102] suggested sericin-loaded cationic gelatin/hyaluronan/chondroitin sulfate electrospun scaffolds. The two GAGs, hyaluronan and chondroitin sulfate, are vital for the development of dermal tissue. Sericin is the main amino acid in the skin with antioxidants, antibacterial, anticancer and UV-light protective properties. Cationic gelatin was selected as the base polymer in the electrospun scaffolds to control the release of the bioactive components (hyaluronan and chondroitin sulfate). Crosslinking of the scaffold using GA significantly improved the degradation time and mechanical properties. They also found that hydrogen bonding between sericin, GAGs and –NH_2_, –COOH functional groups in cationic gelatin prevents chain movement and improves the mechanical properties. In vitro assays proved that both cellular interactions with neighboring cells and with cellular microenvironments could stimulate the differentiation of hMSCs towards epithelial linage. Xu et al. [150] proposed an in situ UV crosslinking technique to strengthen fibers for constructing ECM-mimicking scaffolds for stem cells. As shown in Figure 6e, they embedded PCL fibers into poly(ethylene glycol) (PEG)-fibrinogen (PF), in which CTGF was infiltrated. The scaffold was named PFP-C. An ECM-imitating scaffold was generated by crosslinking of DAs by exposure to UV light. Different studies demonstrated biomimetic ECM nanostructures for homing stem cells along with synergistically facilitating control of stem cells for regenerative treatments. For UV crosslinking of gelatin, Lin et al. [103] proposed phenylazide-conjugated poly(acrylic acids) as a photoinitiator. Under UV radiation, phenylazido groups convert to nitrenes that create permanent covalent bonds with adjacent N–H or C–H. For fortification of the scaffold, they added hydroxyapatite (HA), RGD, and bone morphogenetic protein-2 (BMP-2). Creating interconnections between fibers by crosslinking reagents is categorized into in situ electrospinning and post-spinning crosslinking. Genipin, glyoxal, glutaraldehyde and 1-ethyl-3-(3-dimethylaminopropyl) carbodiimide (EDC)/N-hydroxysulfosuccinimide (NHS) are common crosslinkers for tissue engineering applications. The crosslinking process increases the mechanical features of the scaffolds but leads to the creation of nanofibers with larger average diameters. Moreover, chemical crosslinker residues, due to their cytotoxicity, may reduce cell viability on scaffolds, which can be compensated by the UV crosslinking method.

In addition to the composition of polymers and crosslinking reagents, a wide range of inorganic materials combined with nanofibers led to a positive synergistic impact on the fabrication of scaffolds with better mechanical and morphological properties resembling natural ECMs, which are essentially made of heterogeneous materials. Some of the latest published research is discussed in Section 4.5.

### 4.5. Nanofibers of Polymer–Inorganic Material Composites

**Figure 7:**
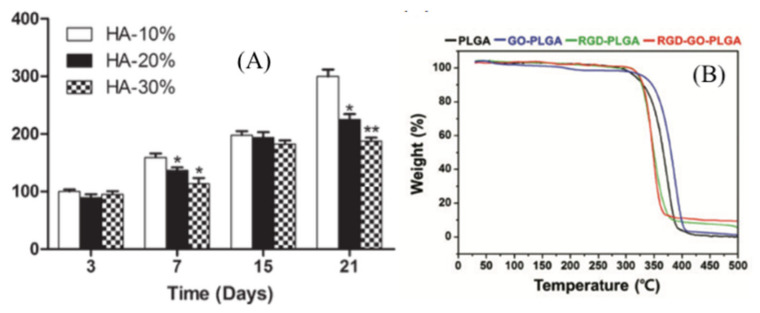
(**A**) Cell proliferation on 10% HA, 20% HA and 30% HA scaffolds performed by alamarBlueVR assay. Duplicated from reference [105] with a permit from Wiley. (**B**) Thermogravimetric analysis of arginylglycylaspartic acid (RGD)– graphene oxide (GO)–poly(lactic-co-glycolic acid) (PLGA). Duplicated from reference [109] with permission from Oxford University Press.

Today, reinforced fibers with inorganic materials have gotten considerable attention, especially in bone tissue engineering. Accordingly, Nagarajan et al. [151] reinforced gelatin by boron nitride and, after electrospinning, crosslinked the nanofibers by GA. They found that the addition of boron nitride to the gelatin matrix effectively improved Young’s modulus. Cell viability tests, fluorescence imaging, osteoblast gene expression and alkaline phosphatase (ALP) activity using human bone cells (HOS osteosarcoma cell line) proved that boron nitride did not affect cell attachment and proliferation. Therefore, boron nitride reinforced gelatin was introduced as a biocompatible and convenient ECM for bone tissue engineering. Pati et al. [104] fabricated chitosan-tripolyphosphate (chitosan-TPP) fibers for imitation of natural ECMs using wet spinning. They mentioned that the immediate ionic crosslinking of chitosan by tripolyphosphate leads to improvement of degradation in consequence of decreased crystallinity. Afterward, in another study, the authors suggested nano/microfibers of collagen mixed with chitosan-tripolyphosphate for bone tissue engineering [152]. Collagen in different ratios was attached to the fibers by a self-assembling method and named Co-Ch-0.1 and Co-Ch-1. The crosslinking process, with the aim of enhancing the mechanical characteristics and stability, was performed using EDC and NHS. In comparison to their previous study, the collagen intermingled chitosan-tripolyphosphate fibers improved the proliferation and attachment of fibroblasts and osteoblast cells [104]. As the bone tissue is principally made of collagen and HA, Vozzi et al. [105] examined the influence of HA percentage and GP crosslinking on features of collagen-gelatin-GP-HA scaffolds for mimicking bone ECMs. They tested cellular adhesion, ALP activity, proliferation, osteocalcin (OC) and osteopontin (OPN) expressions of human primary osteoblasts on collagen-gelatin-GP-HA scaffolds containing 10%, 20% and 30% HA. For all three types of scaffolds, proliferation increased after 21 days, but the highest proliferation was observed for the 10% HA scaffold Figure 7a. After day 3, adhesion and colonization transferred to the inside of the scaffold. Ao et al. [4] prepared and characterized a series of electrospun nanofibers from cotton cellulose and varying contents of nano-hydroxyapatite (nano-HA). The surface analysis demonstrated that by enhancing the nano-HA ratio, the average diameter, strength and thermostability of the fibers increased. Seeding of human dental follicle cells on the fibrous mats showed that coating cellulose with nano-HA improved cell proliferation and did not cause cytotoxicity. With the aim of creating a tissue-like natural matrix, Haj et al. [106] reported a 3D hybrid multilayer scaffold from the combination of osteoconductive ceramic particles and PCL nanofibers. The scaffold supported proliferation and differentiation of hMSC into bone tissue. Different characteristics of the scaffold on micro-and nanoscales were analogous to the ECM of bone tissue.

Although starch, due to its similarity to the cellular milieu, is a noticeable natural polymer in tissue engineering, its weak processability and water uptake restrict its performance [153]. Combining starch with other organic and inorganic materials would be a feasible way to overcome this restriction. For example, Hadisi et al. [107] proposed a novel fibrous scaffold made of silk fibroin (SF) nanofiber-porous starch-calcium phosphate. To fabricate the scaffold, chopped electrospun SF nanofibers were soaked in a starch solution, where glutaraldehyde was applied as a crosslinker reagent. Subsequently, reinforcement of the nanofibers with calcium phosphate was carried out by additional soaking in calcium and phosphate dilutions. The authors found that incorporation of starch decreased the mean pore size, swelling and porosity of the nanofibers. The viability, attachment and spreading of osteoblast-like cells improved on these composite fibers compared to pure starch. In another approach, Nourmohammadi et al. [154] developed a favorable composite for bone tissue engineering. Initially, chitosan-starch composites were prepared by mixing chitosan with various contents of oxidized starch. Subsequently, the prepared and cut calcium phosphate covered PCL fibers were added to the chitosan-starch composite. The authors claimed that by increasing the starch ratio, the porosity and water uptake increased and led to weak mechanical strength, due to a higher amount of hydroxyl groups. Moreover, MTT tests indicated that the viability of osteoblast-like cells (MG 63) improved when increasing the starch ratio. Another study by Wadke et al. introduced starch-based scaffolds consisting of starch, PVA and Ag nanoparticles (NPs), which were fabricated using the electrospinning method. PVA and Ag NPs acted as a plasticizer and antibacterial reagents, respectively. The amount of released Ag NPs from the composite was estimated to be 79.52% after 21 days. The scaffold showed sufficient ability to be used in tissue engineering [108]. In another study, Tan et al. [155] designed nanofibrous scaffolds with blended chitosan, gelatin and shape memory polyurethane. Chitosan and gelatin were incorporated to increase the hydrophilicity and biological features, while shape memory polyurethane was chosen in order to modify the mechanical properties. To enhance the antibacterial properties against Gram-positive and Gram-negative bacteria, the nanofibers were coated with Ag particles by soaking them in an AgNO3 solution for 1 hour. The mean diameter of the formed fibers was reported to be around 300 nm. The membrane had favorable water vapor transmission ratio, mechanical behavior and surface wettability, as well as antibacterial activity, cytocompatibility and hemostatic properties.

Graphene-based materials are known as unique, biocompatible materials due to their outstanding features, such as biocompatibility, thermal and electrical conductivity and biomaterial functionality [156]. Hence, Shin et al. [109] designed RGD peptide and graphene oxide (GO) co-functionalized PLGA (RGD–GO–PLGA) mats. Although it has been proven that the RGD peptide causes better cell attachment and growth, its fabrication process is complicated. To solve this problem, the authors used M13 phages. GO was applied as a nanofiller for promoting thermomechanical features of the scaffolds along with improving cell growth. SEM images showed fibers with an average diameter of 558 nm and a 3D structure similar to natural ECMs. The RGD–GO–PLGA nanofibrous mats had the desired thermal Figure 7b, physiochemical and microenvironmental properties for vascular smooth muscle cells (VSMCs). In another study, Zhang et al. introduced photocatalytic fibers for neural tissue engineering. They spread graphitic carbon nitride (g-C3N4) and GO on the surface of PCL/gelatin fibers and found that g-C3N4 highly improved neuronal differentiation of PC12 cells, while no toxicity was found at concentrations between 0.01 and 0.9 mg/mL [157].

In summary, based on the reported results, additional inorganic materials strengthened the scaffolds in the aspects of mechanical, thermomechanical, antibacterial and cell activity properties. For example, ECMs containing antibacterial reagents such as sericin and Ag nanoparticles were highly applicable in dermal tissue engineering. Boron nitride, hydroxyapatite, tripolyphosphate and calcium phosphate were highly recommended for bone tissue engineering. Graphene-based materials were found to be applicable for promoting thermomechanical features of the scaffolds along with improving cell growth.

## 5. Conclusions

One of the principal objectives of tissue engineering is to repair damaged tissues. To meet this demand, tissue engineers focus on creating ECMs that enables suitable support for cellular function. Nanofibrous scaffolds can properly mimic the ECM multi-fibril network. The electrospinning technique is considered the most applicable method among the various valid methods for the fabrication of nanofibers due to the cost, facility and morphology of the created nanofibers. Multicomponent electrospun nanofibers have frequently been reported to provide ECMs, which are morphologically and mechanically more analogous to native ECMs in comparison to pure polymer nanofibers. On one hand, due to biocompatibility and biodegradation, natural polymers like collagen, gelatin and chitosan are notable, but their unfavorable mechanical characteristics, fast degradation, restrict their applications. From another point of view, synthetic polymers are reproducible and inexpensive; however, they lack biomimetic features.

Here, we first reviewed different scaffolds synthesized from combinations of natural and synthetic polymers. In these cases, all compositions demonstrated better mechanical durability, wettability and cell attachment in comparison to scaffolds composed of one component. Furthermore, all results showed that among films, aligned and random nanofibers, the aligned nanofibers highly support cells and help cells to orientate, thereby increasing cell growth, proliferation and mechanical properties. Generally, besides physical combinations of polymers, natural or synthetic, crosslinking is a strong approach to engineer nanofibers and boost their stability and mechanical properties. Crosslinking can be introduced in situ electrospinning and post-spinning. Genipin, glyoxal, glutaraldehyde and 1-ethyl-3-(3-dimethylaminopropyl) carbodiimide N-hydroxysulfosuccinimide are common crosslinkers for tissue engineering applications. The crosslinking process increases the mechanical characteristics of the scaffolds but often leads to the creation of fibers with larger average diameters. Moreover, chemical crosslinker residues, due to their cytotoxicity, may reduce cell viability on scaffolds. We also discussed how the addition of inorganic materials strengthened the scaffolds in the aspects of mechanical, thermomechanical, antibacterial and cell activity properties. For example, ECMs containing antibacterial reagents, such as sericin and Ag NPs, are highly applicable for dermal tissue engineering.

## 6. Future Perspectives

Future studies should evaluate multifunctional scaffolds that, in addition to physically supporting cell growth, assist tissue regeneration with bioactive signals. In comparison to systemic drug delivery, a combination of tissue engineering and drug delivery methods causes site-specific drug release that boosts drug efficiency, decreases side effects and protects unstable drugs. Last, but not least, as most of the studies were examined under in vitro conditions, further experiments are needed to validate the data in vivo towards clinical applications. Moreover, the interaction of polymeric nanofibers with biological systems that may stimulate inflammatory and allergic reactions should be evaluated.

## Figures and Tables

**Table 1:** Examples of reinforcing polymers and their effect on cell proliferation.

Polymer	Reinforced by	Mean Diameter (nm)	Cell Proliferation			Tissue	Reference
			Unit	Day	Number of Cells		
Gelatin	Silk	83.9	OD_450_	1	1.5	Vessel	[90]
				2	1.6		
Chitosan	Silk	446.9 ± 167	OD_492_	7	1.7	Bone	[91]
				28	2.6		
Chitosan	Gelatin	180	OD_490_	5	0.8	Retina	[92]
				7	1.1		
Gelatin	Tecophilic	409 ± 150	OD_490_	7	0.5	Vessel	[93]
				10	0.9		
Carboxymethyl chitosan	PCL	356	OD_570_	2	0.42	Bone	[94]
				3	0.3		
Gelatin	PCL	330–370	OD_490_	3	0.8	Bone	[95]
				5	1.7		
Chitin	Polyaniline	88.7 ± 19.1	OD_450_	4	1.2	Nerve cardiac muscle	[96]
				7	2.4		
Poly (lactic acid)	Poly (pyrrole)	128.8 ± 27.9	FI	6	290		[97]
				8	380	Nerve	
PLGA	PCL	554	OD_450_	4	0.7	Muscle	[98]
				8	2.2		
PCL	Lignin	259 ± 42	FI	5	460	Nerve	[99]
				10	590		
Gelatin	Tyrosine, glutaraldehyde, 1, 2, 3-triazole ring	350–500	OD_570_	4	0.6	Cartilage	[100]
				7	0.8		
Gelatin	Glutaraldehyde	-	Cell Number (10^4^)	6	3.8	Skin	[101]
				13	7		
Cationic gelatin	Sericin, hyaluronan, chondroitin sulfate, glutaraldehyde	206 ± 45	Cell Number (10^4^)	1	3.7	Skin	[102]
				3	7		
Gelatin	Hydroxyapatite, peptides, UV crosslinking, bone morphogenetic protein-2	-	Cell Number (10^4^)	4	3.3	Bone	[103]
				7	5.5		
Chitosan	Tripolyphosphate	-	Cell Number (10^5^)	5	15	TE	[104]
				7	17		
Gelatin	Collagen, genipin, hydroxyapatite	-	%	7	150	Bone	[105]
				21	300		
PCL	Ceramic	-	FU	7	11,000	Bone	[106]
				21	46,000		
Silk fibroin	Starch, calcium phosphate, glutaraldehyde	-	%	3	125	Bone	[107]
				7	118		
Starch	Polyvinyl alcohol, Ag nanoparticles, glutaraldehyde	110–300	%	7	130	Skin	[108]
				21	190		
PLGA	Graphene oxide, arginylglycylaspartic acid	558	%	5	350	Muscle	[109]
				7	420		

OD, optical density; FI, fluorescence intensity; FU, fluorescence unit.

## Data Availability

No new data were created or analyzed in this study. Data sharing is not applicable to this article.
